# Optical Biosensors for Therapeutic Drug Monitoring

**DOI:** 10.3390/bios9040132

**Published:** 2019-11-11

**Authors:** Vivian Garzón, Daniel G. Pinacho, Rosa-Helena Bustos, Gustavo Garzón, Sandra Bustamante

**Affiliations:** 1 Doctoral Programme of Biosciences, Universidad de La Sabana, Chía 140013, Colombia; 2 Therapeutic Evidence Group, Clinical Pharmacology, Universidad de La Sabana, Chía 140013, Colombia; 3 Faculty of Medicine, Universidad de La Sabana, Chía 140013, Colombia; 4 Physics Department, the Centre for NanoHealth, Swansea University, Swansea SA2 8PP, UK; 5 Vedas, Corporación de Investigación e Innovación, Medellín 050001, Colombia

**Keywords:** therapeutic drug monitoring (TDM), nanobiosensors, optical biosensors, pharmacology, personalized medicine

## Abstract

Therapeutic drug monitoring (TDM) is a fundamental tool when administering drugs that have a limited dosage or high toxicity, which could endanger the lives of patients. To carry out this monitoring, one can use different biological fluids, including blood, plasma, serum, and urine, among others. The help of specialized methodologies for TDM will allow for the pharmacodynamic and pharmacokinetic analysis of drugs and help adjust the dose before or during their administration. Techniques that are more versatile and label free for the rapid quantification of drugs employ biosensors, devices that consist of one element for biological recognition coupled to a signal transducer. Among biosensors are those of the optical biosensor type, which have been used for the quantification of different molecules of clinical interest, such as antibiotics, anticonvulsants, anti-cancer drugs, and heart failure. This review presents an overview of TDM at the global level considering various aspects and clinical applications. In addition, we review the contributions of optical biosensors to TDM.

## 1. Introduction

Therapeutic drug monitoring (TDM) enables one to quantify drugs that have high toxicity by tracking pharmacokinetic changes and determining a narrow therapeutic index (TI). The World Health Organization (WHO) and the United Nations Food and Agriculture Organization (FAO) have stated that the use of drugs requiring TDM has increased during the last few years, mainly due to sanitation and health system-related problems confronting patients and doctors [1,2]. An example of this would be patients suffering cardiac diseases requiring medication involving digoxin, a highly toxic cardiotonic glucoside [3]; people suffering from cancer who are treated with haematotoxic drugs such as paclitaxel [4]; or highly cardiotonic and neurotoxic capecitabine, as well as opioids such as morphine, which have been shown to have a TI [5] because small variations in plasma levels can generate subtherapeutic or supratherapeutic concentrations, leading to adverse reactions in the treatment of patients [6].

Another major problem concerns the accelerated increase of cases with multi-resistant bacteria. This phenomenon has led to the increased use of antibiotics such as colistin, a molecule which had been discontinued due to its toxicity but is now being administered again to patients in intensive care due to the related panorama of antimicrobial resistance (AMR) [7,8]. Doctors are thus obligated to use drugs of last resort, which require a personalized dosage depending on a patient’s condition, which is defined as the patient’s symptoms, signs, and the genetic characteristics of the disease. The above problems are due to the increase in patients with certain pathologies that require special attention by medical staff. An example of this was reported by Papadopoulos and collaborators, who showed that, depending on the resistance of Gram-negative bacteria to antibiotics in addition to the condition of the patients, the use of colistin could be more effective in patients with extensively drug-resistant (XDR) bacteria than in those with AMR, for whom the treatment would be null [9]. Another of the factors that affect the dosage of medications are the genetic characteristics of patients. Recent studies have determined that some genes, such as ABCB1 and ABCC4, directly influence the sensitivity that patients with leukemia can have to chemotherapy with methotrexate, which would lead to a dose modification depending on the patient [10].

The forgoing highlights the task of researchers and people working in the field of health to find alternatives for providing personalized medicine. One such option concerns the monitoring techniques that have led to quantifying these drugs, such as chromatographic methods alone or coupled to masses with a variety of detectors, including ultraviolet or fluorescent detectors (specified below) and immunoassays [11], which are characterized by being very sensitive, possessing high degrees of specific techniques, and being flexible in the analysis of drugs or metabolites. These techniques are mostly approved by the United States Food and Drug Administration (FDA) [12].

However, these time-consuming techniques require specialized laboratories and personnel, with chromatography being one of the most expensive methods when monitoring drugs. This situation sometimes puts monitoring solutions beyond the reach of people working in the field of health and patients. Nevertheless, a class of devices has revolutionized the way drug concentrations in bodily fluids (especially in blood, plasma, serum, and urine) can be measured. These are biosensors, including nano-optical biosensors, which have been developed for monitoring different drugs in a simple, rapid, and inexpensive manner. These sensors have the advantage of being used at a patient’s bedside and being manipulated by doctors or healthcare personnel [13]. They also have other advantages over different techniques, since some of the equipment is low-cost and portable, which makes them a cost-effective alternative when implementing this type of system. Examples include electrochemical sensors and surface plasmon resonance (SPR) made with economic and functional materials [14,15]. This article reviews the use of nano-optical biosensors and their use in TDM to provide data for personalized patient therapy, to minimize any adverse effects (AE), and to enable the safe use of a particular drug. 

## 2. Therapeutic Drug Monitoring (TDM)

The WHO has included specific guideline reports on how a drug should be monitored due to TDM’s clinical importance, which is defined as individualizing a drug’s dose by keeping a drug’s concentrations in the plasma or blood within a target range to act as a guide for healthcare staff [1,16]. Such guidelines deal with large interpatient pharmacokinetic variability, AE and therapeutic concentration-related effects, NTI, undefined therapeutic concentration ranges, and difficult-to-manage desired therapeutic effects. 

This report stressed certain criteria, such as an increased drug concentration in the blood being related to increased efficacy and/or toxicity in the organism, a target drug’s pharmacological effects not being easily monitored, and drug concentration-related AE [1]. Drug concentration at the site-of-action cannot be monitored routinely, but AE can be better correlated with plasma measurements than dosing schemes/schedules. Likewise, this report suggested a list of a pharmacological groups requiring monitoring, i.e., antibiotics (aminoglycosides and glycopeptides), anticonvulsants (valproic acid, phenytoin, phenobarbital, and carbamazepine), cytotoxic drugs (metotrexate), antiarrythmics (digoxin), and immunosuppressants (cyclosporine), which are indispensable drugs for the treatment of a myriad of diseases in current clinical practice [16]. 

**Figure 1:**
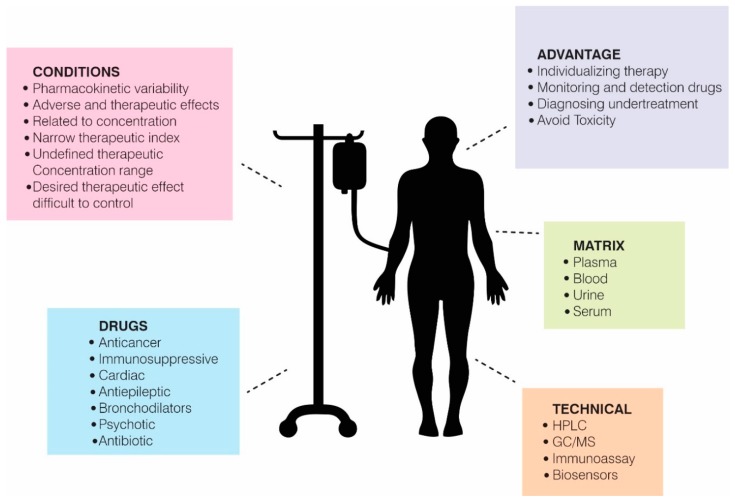
Therapeutic drug monitoring (TDM). TDM is a fundamental tool for the management of drugs with a narrow therapeutic window and high toxicity. For this, different conditions have been established that limit a drug to belong to this group of molecules. These molecules are quantified by different methods, such as HPLC, GC/MS, immunoassays, and biosensors using different body fluids (matrix), with advantages for public and patient health.

In spite of advances in medicine, infection caused by multiresistant bacteria and the use of drugs having a NTI remains a worldwide public health problem. This means that billions of patients in different parts of the world require a solution that provides them with a better quality of life while undergoing treatment for their particular disease. Attempting to address the forgoing, TDM has been used since the start of the 1970s for individualizing pharmacological treatment and keeping a drug’s plasma concentrations within a stated therapeutic range [17]. A particular target drug’s behaviour and the organism’s effects on its absorption, bioavailability, distribution, metabolism, and excretion (pharmacokinetics), and a drug’s effects on the organism, can be evaluated by studying its binding to receptors and chemical interactions (pharmacodynamics) [18] to understand a drug’s behaviour after administration and to thereby determine a drug’s dose and suitable use [19,20] (Figure 1).

TDM has many advantages, making it of great relevance to patients, the scientific community, and doctors, as it enables one to determine the relationship between a formulated drug and its concentration in body fluids. This has led to much patient-related research focusing on personalized medicine and its impact on target populations, with relevant data having been found. These data have facilitated the analysis of emerging diseases and their treatment, i.e., using the highly toxic antibiotic colistin against multiresistant bacteria, which is being used again to combat infections [7]. TDM is useful for determining whether a particular patient is taking the correct drug, as well as for ascertaining interactions with other drugs and food [21]. TDM has become a tool for interpreting measurable values by using mathematical principles, leading to accurate/successful conclusions for optimizing treatment [22,23].

Different techniques have been necessarily employed in TDM due to the nature of the investigated drugs to be quantified in human blood, plasma, serum, saliva, and/or urine. Some of the most commonly used techniques have been high-performance liquid chromatography (HPLC), gas chromatography-mass spectrometry (GC-MS/LC-MS-MS), and immunoassays. The first two are characterized by being the most robust and specific reference techniques; however, these methods require trained personnel, involve long sample processing times, require costly reagents each time the sample is processed in the chromatograph (unlike other techniques that mostly do not require expensive reagents after the validation of the technique), and require a specialized laboratory for processing them, meaning that results cannot be obtained in real time at a patient’s bedside. 

However, these methods have been used for quantifying different molecules in differing matrices due to their robustness, i.e., antibiotics [24,25,26], anticonvulsants [27,28,29], and antineoplastics [30,31,32]. Immunoassays have been of great use as they are techniques that require less time than HPLC and/or GC-MS, as chromatographic techniques require sample preparation time and a mobile phase, extractions and/or filtrations, derivatizations, and continuous control of the equipment for correct operation [12]. However, these techniques require trained personnel and a clinical laboratory with the necessary equipment and reagents. Such techniques include radioimmunoassays (RIA), enzyme-linked immunosorbent assays (ELISA), and fluorescence polarization immunoassays (FPIA), which have been used for quantifying antibiotics, anticancer/antineoplastic, anti-arrhythmic, and biological drugs [33,34,35,36,37,38].

On the other hand, other types of optical techniques have been used for the quantification of drugs, among which are fluorescence-based techniques, which have allowed administered drugs and their metabolites to quantified in real-time, as is the case for SN-38, a cancer drug, in human plasma. With a detection limit of 1.5 ng/mL, this drug is very similar when purchased with results obtained by HPLC [39]. In addition, other types of molecules have been quantified, such as antibiotics like neomycin, which is based on a polydiacetylene system with a detection limit of 2.55 × 10^−7^ M [40].

However, despite the aforementioned techniques providing reliable results, they do have disadvantages when samples have to be transported. Procedures have thus been reported to enable blood samples to be taken more easily and quickly, and without needing to be sent to a laboratory by healthcare staff (i.e., dried blood spot testing). This process consists of collecting a drop of a patient’s blood on a piece of paper suitable to this aim, which is then taken to a laboratory for analysis, mainly by chromatographic techniques [41]. However, the greatest disadvantage of this technique lies in its lack of certainty regarding where and when a particular sample was collected [42]. One of the most innovative techniques available today concerns biosensors, which meet all the aforementioned requirements in a single device. These sensors are sensitive, specific, cost-effective/affordable, portable, do not require a specialized laboratory or trained personnel, and samples can be taken and analyzed at a patient’s bedside by a doctor, thereby enabling faster and more accurate decision making [13]. They thus represent one of the most promising methods for analyzing and quantifying drugs in bodily fluids in advance personalized medicine.

## 3. Biosensors

Biosensors have become a fundamental tool in different fields of the global economy, arousing great scientific and industrial interest, since they represent an alternative to traditional analysis methods, with properties such as high detectability, specificity, short analysis time, integration with portable systems, easy automation, real time functionality, versatility, and low cost [43]. These properties give multifunctional biosensor devices different applications in medical diagnosis [44,45,46], biopharmaceutical applications [47,48], immunoassays [49,50], food analysis [51,52], biomarker determination [53,54], screening drugs [55,56], and tissue engineering [57,58,59].

According to the International Union of Pure and Applied Chemistry (IUPAC), the term biosensor is defined as “an independently integrated receptor transducer device, which is capable of providing selective quantitative or semi-quantitative analytical information using a biological recognition element” [60]. A biosensor consists of an analytical device incorporating a biological recognition element intimately associated with or integrated into a transductor. A variation of the physical–chemical properties recorded by the transductor as a product of the interaction between the analyte and the biological element provides a quantifiable signal that can be amplified and processed and is proportional to the concentration of analyte to be analyzed [13]. Biosensors can be classified according to the nature of the biological components and transduction system being used.

Biological components are classified as catalytic or affinity biosensors. The former are used in systems containing isolated enzymes incorporated into cell organelles, complete cells, or tissue; these sensors are mainly based on the chemical reactions catalyzed by an enzyme, with the substrate measuring the reaction products from such interactions [44]. Affinity bioreceptors are based on the interaction between the analyte and the recognition element, forming an analyte–receptor complex, which is detected by labelling (enzymatic or fluorescent) or by a change in some of the transductor’s physical–chemical properties [13]. Some of the most commonly used receptors are antibodies (Ab), nucleic acids, microorganisms, aptamers, and receptor proteins. The transduction system converts the recognition element into a signal measurement, thereby enabling biosensors to be classified into electrochemical (amperometric, potentiometric, and impedimetric), optical (fiber-optic, evanescent wave, and SPR), piezoelectric (quartz crystal microbalance (QCM)), and nanomechanical (nanocantilevers) [61]. 

A combination of bioreceptors and transductors forms a fundamental mechanism when developing a biosensor device [62]. Electrochemical biosensors are characterized by measuring the change in currents and voltages produced in the medium as a consequence of a molecular recognition type reaction, this change being proportional to the concentration of an analyte to be determined [60]. The obtained signals are classified as potentiometric, impedimetric, and amperometric [63]. Potentiometric biosensors include a selective electrode, a reference electrode, and a device that measures potential changes. The selective electrodes are made up of conductive materials, which allow the quantification of the analyte that is immersed in a solution, allowing the measurement of changes or variations in the potential [40,64]. Impedimetrics record changes in the environment by monitoring the impedance between electrodes or disturbances at the electrolyte/electrode interface [65]. Amperometric biosensors measure current flows generated by an electrochemical reaction at a constant potential, where the current intensity is directly related to the concentration of the substance oxidized and reduced on the electrode’s surface [63]. These types of biosensors have been used in quantifying aminoglycoside antibiotics [66], bronchodilators (such as theophylline) [67], anti-arrhythmic [68], and anticancer drugs [69,70,71,72,73,74,75,76,77,78,79,80,81,82,83].

Piezoelectric biosensors are characterized by having an oscillating piezoelectric crystal resonating at a natural resonance frequency; this frequency is controlled by a signal giving an electric current value. Thus, when the analyte comes into contact with the detection material it causes a displacement in vibration frequency, thereby producing changes in the electric current being recorded, which is directly related to the analyte of interest [84]. These biosensors are considered very useful tools for measuring analytes related to quality control in industry [85] and have been a fundamental tool in a clinical settings for quantifying immunoglobulins and insulin [86], methamphetamines [87], and cocaine [88] (Table 1).

**Figure 2:**
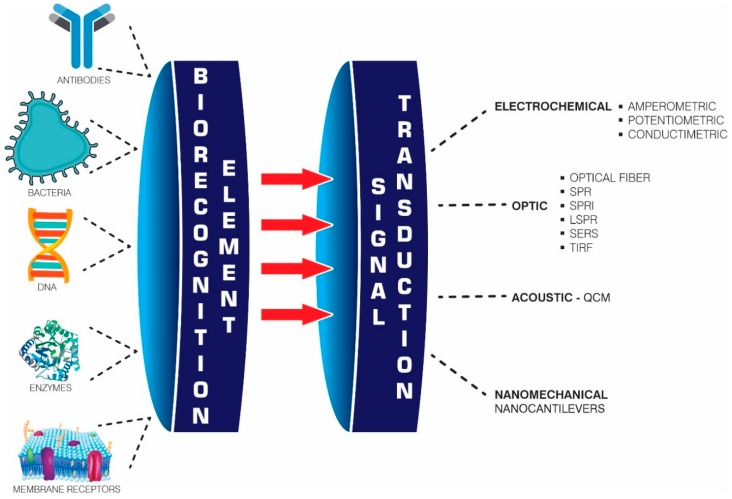
Scheme of a biosensor. Biosensors are made up of a biological recognition element and a method of signal transduction, and have allowed the quantification of various analytes.

Nanomechanical biosensors are based on cantilever flection when a molecular interaction occurs on their surface; this molecular recognition becomes nanomechanical movement, which is coupled to an optical or piezoelectric system [89]. These cantilevers are usually made from poly-silicon, silicon nitride, silicon oxide, or polymeric materials, in which the cantilever beams are covered with a sample that has been exposed to analyte vapor [90]. Studies by Fritz et al. demonstrated nanomechanical sensors’ ability to differentiate variations in a single base in the DNA chain without using fluorescence markers. They have also been widely used in evaluating proteins, such as topoisomerases [91] or proteins related to prostate cancer [92], as well as in detecting antibiotics for combating Escherichia coli [93] (Table 1). Optical biosensors have been used very recently due to them having been markedly improved in their microfluidics and imaging systems. Further, they are label-free, depending on the analyte, and are quantified in the femtogram range, making them promising devices for quantifying drugs requiring TDM. Figure 2 lists the main characteristics of these devices according to their physicochemical principles.

## 4. Optical Biosensors

Optical biosensors are devices that detect changes in the properties of light, such as the refraction index, absorption, fluorescence, or light scattering resulting from the interaction between an analyte and a receptor [117]. This produces a signal proportional to a substance’s concentration, measured using biological materials (including enzymes, Abs, antigens, receptors, nucleic acids, cells, and complete tissue) as biorecognition elements [118]. Optical biosensors have great advantages as some of the best sensors in cataloguing affinity or catalytic receptors. They have greater sensitivity and versatility, which thus enables faster and real time measurements, and can be adapted for multichannel and multiparameter detection [119].

These devices have been used for monitoring NTI drugs, i.e., antibiotics of last resort involving risks of toxicity in patients. One of the most commonly used biosensors has been the SPR biosensor, which has become an alternative in clinical diagnosis due to its capacity for the real-time detection of molecules [120]. Amikacin is one of the molecules that have been quantified by SPR. It is an antibiotic of last resort used mainly for treating infections involving gentamycin- and tobramycin-resistant Gram-negative microorganisms, producing extremely serious secondary effects such as nephrotoxicity and ototoxicity. SPR has enabled rapid and sensitive quantification in plasma [121]. 

Biosensors can also be used for quantifying antineoplastic drugs that produce serious secondary effects that cause toxicity in patients. However, these drugs are not monitored regularly due to a lack of real-time solutions and the low cost of using them at a patient’s bedside [122,123,124]. Metotrexate and mitoxantrone are currently being monitored, as they are widely used for treating lung and breast cancer, leukemia, and lymphoma (despite having high toxicity) and have been quantified in human serum [125,126,127]. More is written about these applications later in this study.

### 4.1. Classifying Optical Biosensors

Optical biosensors can be grouped into two categories, as follows: Bio-optrodes and evanescent field-based ones. Bio-optrodes are based on the interaction between an analyte and an immobilized reagent in the exit of a fiber, which produces a quantifiable change in the transductor’s optical properties. This change is optically evidenced by active groups like dyes, fluorescent molecules, and bio- or chemo-luminescents [128]. Evanescent field-based biosensors are based on electromagnetic waveguides that transmit light by multiple internal reflections in total reflection conditions, thereby making an evanescent field capable of penetrating internal reflections at a determined distance from the waveguide surface, modified by the receptor [118]. Optical evanescent wave biosensors are the most numerous and are characterized by involving the use of some type of electromagnetic field and the principle of ideal evanescent field detection for measuring any biochemical reaction taking place within it, thus making them indispensable tools for analyzing and identifying chemical or biological substances with a high degree of sensitivity and selectivity [129]. Fibre-optic devices, therefore, belong within the bio-optrode biosensor category, while evanescent field devices include SPR-based, surface-enhanced Raman scattering (SERS), total internal reflection fluorescence (TIRF), optical waveguide interferometer, and elipsometric and reflectrometric interference spectroscopy (RlfS) biosensors. 

#### 4.1.1. Fibre-Optic Biosensors

Fibre-optic biosensors (optrodes) are devices in which a biocatalyzer is immobilized at the distal end/tip of a fibre-optic detection device. A biocatalyzer mediates between a sensor and an analyte, forming a detectable compound from a sample of interest [130]. This type of biosensor has been studied for monitoring cells in clinical samples, endotoxins produced by Staphylococcus aureus, Clostridium botulinum [131,132], and proteins with clinical relevance, such as cardiac markers and anticoagulants [133].

Using this type of biosensor to quantify drugs has gradually increased in prevalence. An example of this would be the reports in the pertinent literature concerning molecules such as phenytoin, an anticonvulsant that is widely used in clinical practice. A high level of efficiency was found regarding its sensitivity compared to the reference method (gas chromatography), which has a 4.45 µM detection limit at 37 °C. It is worth stressing that variables like temperature and pH were evaluated in the study as they affect equipment functionality [134]. This type of device has also been used to measure theophylline, a drug used for treating respiratory symptoms associated with diseases like asthma, chronic bronchitis, and emphysema. Abs have been used to determine analyte concentrations, leading to a change in the Ab binding equilibrium/balance between labelled theophylline and unlabeled theophylline, provoking an increase in fluorescence. Detection ranged from 55 to 110 µM in human serum [135].

Optrodes have been used in sectors such as agriculture to quantify organophosphorus pesticides using the principle of conical-shaped optical fiber detection. This form of fiber facilitates better evanescent field interaction, providing greater sensitivity and specificity. A 2.4 × 10^−10^ M limit for detecting methyl-parathion (a prohibited organophosphate due to its high toxicity indices for human health and the environment) makes these devices extremely useful for determining pesticide concentrations worldwide, which have caused intoxication and death in animals, plants, and human beings [136].

Scientists have modified this technique to obtain better results, calling this biosensor a “bioluminescent-based fibre-optic biosensor” based on its cells’ capacity for continuous monitoring of their microenvironment and responses to environmental changes when expressing specific genes [8]. Cellular responses thus become signals that are detectable by a sensor. Genetically modified live cells are used for this as they can emit a bioluminescent signal that is detectable by an analyte. Such cells have previously been immobilized in optical fibers arranged in a series of high-density microwells [118]. This type of biosensor has mainly been used for detecting genes; for example, Biran et al. demonstrated the efficacy and sensitivity of this type of sensor in determining the presence of genotoxic agents using a genetically modified E coli strain, using a luminescent signal as their experimental model [118,137].

#### 4.1.2. Surface Plasmon Resonance (SPR)-Based Optical Biosensors

SPR biosensors are new technologies that are widely used due to their greater sensitivity and simplicity and ability to provide real-time results [138]. This phenomenon was observed for the first time and reported in the pertinent literature by RH Ritchie (1957), who demonstrated that there was a loss of energy when the electrons penetrated metal, giving rise to the concept of “metallic plasmon” for describing fluctuations in the internal density of metal electrons [139]. The term plasmon was derived from the concept of plasma, due to them both being constituted by charged particles collectively responding to a stimulus. Plasmons are defined as a quantum of energy (i.e., plasma oscillation) associated with wave propagation in material via the collective movement of a large amount of electrons, phonons, or photons [140], in which free electrons respond by collectively oscillating in resonance at the same frequency as the incident light. Such oscillations are known as plasmon surface (PS) resonance [117].

The SPR phenomenon is based on an optical measurement of refractive index changes using monochromatic light that excites the plasmons. The measurement involves immobilizing the recognition element over the surface of a metal and placing it against a prism from the equipment’s optical system. The light from a polarized infrared light emitting diode (LED) is focused via the prism onto the metal surface, such that the incident light beam becomes dispersed, giving a range of incident angles [141]. The light reflected from the metal is detected by the photodiode matrix covering an appropriate interval of refraction angles. The plasmons are excited at a determined incident angle, and the corresponding loss of beam potential reflected at such an angle is recorded [140]. The incident angle depends on many factors, such as the refractive index close to the back part of the metal film, where the molecules were previously immobilized, and the chemical nature of the molecules [139].

These types of biosensors are considered universal detectors, as they can detect a large amount of molecules. However, the reduced size of some molecules in therapeutic drugs does not affect the refraction index, meaning that they cannot be detected on some occasions. Indirect techniques, such as competition assays and secondary detection with functionalized Abs and nanoparticles (NPs) have thus been introduced to increase the sensitivity of these biosensors.

SPR approaches have been at the forefront of clinical research, mainly in quantifying drugs requiring TDM, such as antibiotics, anticancer drugs, and anticoagulants. Regarding antibiotics, vancomycin and chloroeremomycin have been quantified by covalently coupling bacterial cell wall peptides to an HS(CH_2_)_15_CO_2_H self-assembled monolayer (SAM) on a gold film. The vancomycin detection limit was found to be 20 ± 0.31 mM and 2.5 ± 0.04 mM for chloroeremomycin, thereby showing that these antibiotics (especially chloroeremomycin) are related to bacterial cell wall peptides, enabling better binding to the monolayer and improving quantification [142,143]. Ciprofloxacin has been monitored by an SPR biosensor based on a molecularly imprinted polymer (MIP); this modification has been of great use in in the food industry and the medical field, facilitating the selective detection of antibiotics. It had a ~0.08 μg/L detection limit, which is lower than that reported in the literature, thus making it a more sensitive technique for quantifying molecules of this type in different matrices [144].

Tomassetti et al. worked on the direct detection of ampicillin using an SPR operating in flow conditions. This proved to be more selective than other biosensors when compared to antibiotics with similar structures, but less sensitive than biosensors lacking such modifications (10^−3^ M to 10^−1^ M detection range) [145]. Another modification providing advantages for this type of biosensor is based on Laser Doppler Micro-electrophoresis with UV-Visible (UV-vis) spectroscopy, used to detect gentamycin. This enabled the researchers to determine a 0.05 ng/mL detection limit, which is lower than that for ELISA techniques (0.1 ng/mL limit), making it one of the best alternatives for detecting this type of antibiotic [146]. 

Neomycin B is another antibiotic that has been monitored with aptamer-based biosensors. High sensitivity has been found, with a detection range from 10 nM to 100 µM, thus highlighting the feasibility of the high sensitivity detection of small molecules using RNA fragments [147]. Amikacin has been one of the most reported molecules. Amikacin is an antibiotic that is mainly used in neonates but is suggested for TDM due to its high toxicity, which is inherent in its use. The forgoing has meant that adult and neonate sera have been evaluated by SPR-based indirect competition immunoassays, finding high levels of specificity and sensitivity (1.4 ng/mL 50 CI) and a 0.13 ng/mL detection limit, thereby allowing this drug to be quantified in real-time [120,121]. 

Regarding anticancer molecules, levels of a highly cytotoxic drug called metotrexate (MTX) have been recently measured in the serum of chemotherapy patients using folic acid-functionalized gold nanoparticles (FA-AuNPs) in assays with MTX. It had a 28 nM detection limit [125], which is lower than that previously reported by the author (155 nM), quantified by an LSPR (localized surface plasmon resonance) biosensor [127]. This makes SPR a highly sensitive technique for detecting certain antineoplastics. Simultaneously, studies have been carried out using the interaction of doxorubicin (DOX) with electrodes in different types of biosensors, including SPR. DOX is an antineoplastic, which is commonly used in chemotherapy, despite being cytotoxic. It was found that the monolayer’s hydrophilic and hydrophobic properties are fundamental for proper functioning of the device [148]. Such studies enable procedures to be standardized, thereby facilitating correct functioning when quantifying a target drug (i.e., for TDM purposes).

Some anticoagulants are characterized by having an NTI, and their pharmacokinetics and pharmacodynamics depend on a particular patient’s conditions where high or low doses could cause death or irreparable damage to some organs. An example of this would be quantifying heparin in plasma samples with a 0.2 U/mL detection limit and using protamine and polyethyleneimine (PEI) as heparin affinity surfaces [5,124,149]. Studies have also been carried out to quantify opioids, such as morphine-3-glucuronide, in urine (i.e., M3G is the main metabolite in heroin and morphine). This process involved immunoassays using polyclonal Abs from New Zealand rabbits (Enterprise Ireland and Science and Technology Against Drug Initiative, Dublin, Ireland). Two types of Abs were obtained in the following detection ranges, as follows: From 762–24,400 pg/mL (Ab 1) and 976–62,500 pg/mL (Ab 2). It was concluded that using biosensors is a sensitive technique for detecting opioid analgesic drugs [150].

In addition to this type of biosensor’s advantages and numerous applications, there are some variations, such as SPR imaging (SPRi) and localized surface plasmon resonance (LSPR). SPR imaging (SPRi) combines SPR sensitivity with spatial imaging, thereby enabling multiple interactions to be studied simultaneously. It is characterized by having high performance, high sensitivity, and the ability to obtain images of biointeractions [151]. The pertinent literature contains few reports about TDM and this type of biosensor. These types of sensors have mainly been used for quantifying metaloproteinase-2, a relevant enzyme in angiogenesis, wound healing, and tumor cell metastasis [152]. This type of biosensor offers so many advantages that recent studies have incorporated a smartphone into the system to obtain real-time results when taking measurements, without the need for sophisticated or large volume-occupying equipment, such as a computer [153].

Localized surface plasmon resonance (LSPR) is based on the collective oscillation of free electrons within metallic NPs (gold and silver), where the energy essentially depends on the form and size of the NPs [85]. This technique is highly sensitive, especially in the field of diagnosis, for detecting diseases identified by biomarkers, such as proteins [154]. It has also been used in measuring MTX, an anticancer drug, by using NPs or FA-AuNPs. This device was made for detecting nanomolar to micromolar concentrations of a target drug in plasma (a 155 nM detection limit was eventually found). Measuring this drug enabled the monitoring of MTX levels in patients undergoing chemotherapy [127]. 

Tobramycin is one of the antibiotics being monitored by this type of biosensor. It is a molecule with harmful secondary effects that cause nephrotoxicity, cochlear and vestibular toxicity, ototoxicity, and neuromuscular blocking. Studies reported in the literature have quantified this drug by transmission localized surface plasmon resonance (T-LSPR). This process includes antibiotic-specific DNA-aptamers. Its high sensitivity and specificity were determined, with a detection limit of 0.5 μM [155]. Caglayan and Onur made another type of modification to this type of biosensor, which involved a colorimetric detection technique using silver NPs showing the interaction between negatively-charged particles and cationic aminoglycoside antibiotics and visually indicating a change from yellow to red in the presence of gentamycin, tobramycin, and amikacin. The detection ranges were 20 to 60 ng/mL for gentamycin, 23 to 60 ng/mL for tobramycin, and 60 to 100 ng/mL for amikacin [156,157].

Other types of drugs have been quantified, such as an anticoagulant called megalatran. This drug was monitored by LSPR integrated into a microfluidic lab-on-a-chip device. This process involved immobilizing human α-thrombin on the biosensor’s gold surface for the enantioselective detection of the drug’s enantiomers (0.9 nM detection limit), this being one of the pioneering studies regarding the use of enantioselective biosensors [158]. Studies are currently being advanced for quantifying acenocoumarol (Sintrom), an oral anticoagulant. These studies involve using an LSPR-based nanoplasmonic biosensor alongside highly specific polyclonal antibodies with a 0.66 nM detection limit, which has been catalogued as being a relevant limit for quantifying these drugs [159]. 

This method has also been used for detecting, in serum, drugs that have been used to control arrhythmias and cardiac problems; DOX is one such example, as it has an NTI, but few studies have been made on its dose/administration and high toxicity. This technique consists of LSPR quantification using gold NPs (2 ng/mL detection limit), thus making it sensitive and effective for quantifying this type of molecule [160].

#### 4.1.3. Surface–Enhanced Raman Scattering (SERS)

SERS are based on amplifying the intensity of the Raman phenomenon by using NPs or metallic structures. The electromagnetic field becomes drastically amplified when two particles come into contact with each other, and one of them has rougher material on its surface, resulting in a large amplification of Raman scattering [11]. This type of biosensor has been of great use in quantifying drugs; examples include using silver NPs for antineoplastics, such as MTX, or drugs such as folic acid, as the detection limit for folic acid and MTX is 100 pM—a lower detection limit compared to that found in other studies focusing on these molecules [161]. 

A drug quantified by this type of biosensor is 5-fluorouracil (5-FU), which is used as an anticancer drug in combating a wide range of cancers in the colon, rectum, breast, and head. SERS is thus used with silver NPs to measure drug concentration in saliva. Its 150 ng/mL detection limit is lower than that obtained in other studies. Such research has enabled a correct drug dose to be administered, as its action and toxicity vary from patient to patient [162]. 

This technique has been used to detect antibiotics, finding 27 ng/mL for ampicillin, 29 ng/mL for penicillin G, 30 ng/mL for carbenicillin, and 28 ng/mL for penicilloic acid when using hydroxylamine gold NPs. This offers a promising methodology when detecting penicillin-related antibiotics, providing a new index for quantifying antibiotics [163].

#### 4.1.4. Total Internal Reflection Fluorescence (TIRF) Biosensors

TIRF biosensors are based on using fluorescent molecular markers where evanescent field radiation is absorbed by a probe immobilized on the waveguide’s surface, thereby inducing its fluorescence. Such emission intensity is measured and related to the concentration of the analyte in a particular sample [164]. TIRF techniques give better results than direct detection techniques. Some of their advantages are related to having greater specificity regarding a molecule of interest, as their response is not affected by a sample’s components, meaning that they have been used on liquid samples such as wastewater, sewage, and/or plasma. Fluorescent probes have greater stability than the enzymatic components used in other biosensors and usually have a longer shelf-life and stability than radioactive probes, making them safer [165]. 

These biosensors have thus been widely used in different areas, especially for the environmental monitoring of wastewater from industries, which could affect human and animal health [166]. Ehrentreich-Forster et al. have described using fluorescence biosensors for detecting explosives, toxins, narcotics, and other compounds prohibited by law, which is of great help in controlling illegal substances [167].

This type of biosensor has been of great use in quantifying immunosuppressant drugs due to their narrow therapeutic ranges, where high levels can cause secondary effects and low levels can increase the risk of rejection. Mycophenolic acid (MPA) or mycophenolate mofetil (MMF) using sheep and donkey polyclonal Abs have been reported in the literature. It has been found that this device can detect the drug, thereby providing a great advancement in personalized medicine for patients undergoing transplants [168]. They have also been used for quantifying antithrombin using immobilized heparin [169]. 

Other types of optical biosensor-related devices have been used for quantifying other types of molecules that are important in medicine and industry, such as optical waveguide interferometer biosensors, which are based on combining evanescent field detection with methods for measuring phase difference [170]. This technique has been useful for detecting cell content redistribution, taking cell responses and processes into account, such as detecting the avian flu virus [171]. Elipsometric biosensors are based on changes in the polarization of light when reflected off a surface. These are mainly used in detecting tumor biomarkers or the influenza virus [172]. Reflectometric interference spectroscopy biosensors are based on changes in the phase and amplitude of polarized light, thereby providing information about a protein’s refraction index [173]. This method is used for detecting cancer cells and quantifying contaminants in milk [174]. 

The use of optical biosensors for TDM at the patient’s bedside poses a challenge for researchers because it requires portable devices that guarantee high specificity, sensitivity, speed, and low cost, with the transmission of new materials and technologies that monitor in real time [175]. This type of biosensor uses different bodily fluids, among which are mainly sweat, tears, saliva, and urine.

Among the portable optical biosensors used are biosensors based on contact lenses for the quantification of glucose in tears. These devices are made of a selective glucose hydrogel film functionalized with phenylboronic acid. Evidence shows a sensitivity of 12 nm/mM and a saturation response time of fewer than 30 min. This biosensor is compatible with smartphones, so patients can see the results in real time [176]. On other hand, using dermal biosensors to control glucose levels is a novel technique that allows the measurement of blood levels to be a non-intrusive technology for patients. This new method is based on a band that is placed on the wrist; through combined near-visible infrared spectroscopy (Vis-NIR), it allows the measurement of glucose found mainly in arterial blood [177].

This new technology applied to optical biosensors has allowed the quantification of some drugs, such as phenytoin, a salivary antiepileptic using a portable handheld SPRi with a detection limit of 50 nM; obtaining results in less than 5 min. The measurements of this device were approximately 25 × 10 × 5 cm^3^ [178]. Another example of portable biosensors is those used for the measurement of antineoplastic agents, such as tamoxifen (TMX), using a four-channel portable LSRP. This biosensor allows for high sensitivity (5 nM) because it uses gold nanoparticles and allows quick reading in less than 5 min [125]. Cappi et al. have shown that the measurements of serum tobramycin levels with LSPR are the size of the palm of one hand. The biological components were aptamers functionalized with gold nanoislands (NI) deposited on a glass slide covered with fluorine-doped tin oxide. The detection limit was 3.4 µM. [155].

However, the use of portable biosensors in TDM is very low, as these biosensors are mainly used to determine the concentrations of different pollutants in environmental samples. An example of this is the research conducted by Shriver et al, which determined the presence of a trinitrotoluene (TNT) explosive using a portable fiber optic biosensor [179] or the use of biosensors for the quantification of organic contaminants in water and food by optical immunosensors [180,181]. On the other hand, research has been carried out focused on the determination of antibiotics in milk to preserve the quality of the food, as is the case of the quantification of fluoroquinolone residues using SPR [182]. 

Considering the studies described above, a new window of possibilities for the implementation of portable biosensors in TDM has been opened. This will allow us to find new methodologies to determine and quantify different molecules in body fluids. It is important to keep in mind that there may be different variants according to the nature of the molecule and the matrix used so they can guarantee the development of optical biosensors.

Optical biosensors provide a great tool that has enabled new technologies to be advanced in the area of personalized medication using nanotechnology in different fields of medicine. Table 2 lists the most relevant studies that have involved using different types of optical biosensors.

## 5. Surface Functionalization in TDM

One of the most important aspects when performing TMD using biosensors is the choice of a solid surface and the development of a suitable chemical surface [183]. As TDM is performed in serum or plasma media, where countless proteins are found, it is important to keep the sample intact and free from degradation products. On the other hand, TDM should be used for the ligand where proteins, integrity, native conformation, and functionality are normally important [184]. The chemical selectivity of the functional groups of proteins that are directly immobilized to the analyte to be detected must be controlled by the surface density, uniformity, and non-formation of the artefacts on the sensor’s surface. To choose the correct functionalization of the sensor surface, three important aspects must be considered, as follows: (i) The type of surface, (ii) the type of ligand, and (iii) the type of analyte. For the first case, there are several surfaces or biosensing chips based on printing methods, such as thermal jet inkjet printers (with and without modifications) [185] and the deposition of inert metals such as gold, platinum, and others functionalized by a chemical cover, such as carboxymethyl dextran, streptavidin, Protein A, and lipophilic modification, among others.

Once the appropriate sensor surface has been chosen, some of these surfaces should be functionalized as gold surfaces to improve biosensor sensitivity and minimize non-specific interactions. According to aspect two, the nature and structure of the ligand will define the best way to modify the surface (e.g., wet chemical, organosilanization, ionized gas treatments, and UV irradiation [186]). There are different physical and chemical methods to functionalize surfaces. The physical methods will allow a surface change in terms of roughness, removal of grease and impurities, and surface pore sizes. The foregoing will facilitate the greater adsorption of biomolecules or immobilization through chemical method stability for analytical measurements. Likewise, chemical methods must allow a strong and long interaction between the sensor’s surface and its structure. The functionalization of the surface must allow, in some cases, the orientation of the molecules (antibodies). For the above, coupling covalent, amine coupling, disulfide based, affinity capture, biotin-avidin based, nickel-nitrilotriacetic acid (Ni-NTA) based, antibody-based, and protein A or G based and other molecules can be used. Finally, for aspect three (the type of analyte), numerous studies for TDM involve small molecules of synthetic origin, such as antibiotics, anti-neoplastics, and anticoagulants that differ from large molecules as biopharmaceuticals.

Based on the previous panorama involving different elements, surface functionalization has been used for TDM. Ranamukhaarachchi et al. evaluated Vancomycin levels using a microneedle-optofluidic biosensor functionalizing streptavidin-biotin due to its high affinity, binding, stability, and adaptability to various chemical methods. Biotin binds to assembled monolayers (SAM) of methoxy polyethylene glycol-thiol (mPEG-SH) and acts as a gold sensing surface. This detection system allowed us to evaluate vancomycin with a high sensitivity (0.41 AU/decade) and a low LoD (84 nM) in clinically relevant ranges (from 0.3 to 40 μM) for an extremely low volume (0.6 nL), as well as to perform rapid measurements (<5 min in total) [187]. Other studies to measure methotrexate, testosterone, and antibiotics using SPR used functionalized chip surfaces to evaluate these drugs in biofluids [188].

Most of the measurements for TDM using biosensors are for small molecules. However, the use of optical biosensors (SPR) for plasma levels in biopharmaceuticals has begun to develop in recent years. Such is the case for the measurement of the plasma levels of antibodies and anti-drug antibodies for infliximab (IFX) [189] and anti-TNF-alpha [35]. For the determination of these serum levels, sensor surfaces were used for activation via amino coupling sulfo-N-hydroxysuccinimide/1-ethyl-3-(3-dimethylaminopropyl)-carbodiimide (sulfo-NHS/EDC). The results obtained were reproducible in concentration ranges of 1.39 to 4.76 ± 0.03 µg/mL for IFX and 0.6 to 1.0 µg/mL.

## 6. Conclusions

The growing demand for drugs having an NTI has been partly due to the increase in antibiotic-resistant bacteria (ARB) and the increasing amount of cardiovascular and nervous system diseases that have triggered the use of highly toxic molecules in the doses currently being prescribed. Optical biosensors are thus one of the most promising solutions for this scenario as they play an important role in quantifying drugs and represent one of the most specific, sensitive, low-cost, and easy-to-use options currently available. This has created a niche for them as new clinical tools that enable the therapeutic monitoring of drugs aimed at more personalized medicine by minimizing complications or secondary effects as far as possible and leading to safer patient recovery and medication.

Furthermore, using biosensors provide doctors with an advantage when making the most accurate decisions for real-time dosage at a patient’s bedside as biosensors are easy-to-use and miniaturized portable devices. They provide stricter and more successful control when formulating drugs. Further studies focusing on quantifying drugs in blood and covering all pharmacological groups are urgently needed to ensure that a larger number of drugs have personalized doses in line with the premise of using nanotechnology as a tool for controlling twenty-first century diseases, particularly ARB-related ones.

Some aspects of nanomaterials, such as sensor surfaces constructed from metals like silver, represent limitations in the evaluation of TDM with optical biosensors. It has been observed that nanoparticles made of this material could be released from their surface by general oxidation to biofluids [190]. It is important to note that the development of nanomaterials for the construction of optical biosensors is a great challenge, such that these nano-scale particles cannot contaminate plasma samples from patients in a state where the determination of MDD is vital for a favourable clinical outcome. The protocols established for the measurement are fundamental.

## Figures and Tables

**Table 1:** Biosensors’ general characteristics.

Biosensor	Measurement	Graph	TDM Applications	Ref
**Electrochemical**				
Amperometric	Measuring current flows produced by an electrochemical reaction	Amperogram	Morphine, Metotrexate, Gentamycin, Tamoxifen, Gemcitabine, Didanosine, Irinotecan, Cyclophosphamide, Ifosfamide, Ftorafur, Etoposide	[69,70,73,75,76,94,95,96,97]
Potentiometric	Measuring variations potential (VP) on the electrode’s surface	Potentiogram	Diclofenac, Penicillin, Tetracycline, Flucloxacillin, Doxycycline, Methotrexate, Cisplatin, Titanocene dichloride	[79,98,99,100,101,102,103]
Field-effect transistor-based biosensor (FET)	Measuring variations in the current on the sensor’s surface	Time vs. current	Penicillins, Tenofovir, Procaine, Heparin, Imatinib	[82,104,105,106,107,108]
Impedimetric	Measuring changes via the impedance between electrodes or the perturbation caused by electrolytes/electrodes	Impedance graph	Neomycin, Penicillin, Ciprofloxacin, Bleomycin, Mitomycin C	[65,71,72,109,110,111]
**Piezoelectric**				
Quartz crystal microbalance (QCM)	Measuring the vibration frequency and displacement producing changes in electric current	Frequency variation	Penicillins, Sulfamides, Diazepam	[87,112,113,114]
**Nanomechanical**				
Nanocantilevers	Measuring the cantilever flexion when a molecular interaction occurs on a surface that becomes a nanomechanical movement	Time vs. deflection	Paclitaxel, Vancomycin	[89,115,116]

**Table 2:** Therapeutic drug monitoring using optical biosensors.

Type of Biosensor	Type of Drug	Drug	Biosensor Characteristics	Matrix	Limit/Detection Range	Results	Ref.
**Fibre optic**	Anticonvulsant	Phenytoin	Autonomous reversible immunosensor: mouse monoclonal IgG	Blood and plasma	4.45 µM	Viability for quantifying phenytoin in blood, applicable for other haptens in blood	[134]
	Bronchodilator	Theophylline	Fluorescence-based autonomous reversible immunosensor: mouse monoclonal IgG	Serum	55 µM	Analyte concentrations give rise to a change in the antibody binding equilibrium with changes in fluorescence	[135]
**SPR**	Antibiotic	Vancomycin and Chloroeremomycin	Covalent bacterial wall peptide coupling to a self-assembled monolayer (SAM) on a gold film	Solution buffer	20 mM 2.5 mM	Chloroeremomycin is related to bacterial wall peptides, thereby facilitating quantification	[142,143]
		Ciprofloxacin	SPR with a molecularly imprinted polymer (MIP)	Solution buffer	0.08 µg/L	Sensitive technique for quantifying this type of molecule	[144]
		Ampicillin	SPR operated in flow conditions	Solution buffer	10^−3^ M to 10^−1^ M	Technique requiring less time (20 min) without losing sensitivity	[145]
		Gentamycin	SPR with a Doppler laser using UV-Vis spectroscopy	Solution buffer	0.05 ng/mL	Lower detection limit compared to ELISA	[146]
	Anticancer	MTX	LSPR with functionalized gold nanoparticles with folic acid (FA-AuNPs) in completion with MTX	Serum	28 nM	Lower detection limit than that reported for LSPR biosensors (155 Nm)	[125]
	Anticoagulant	Heparin	Using prolamine and polyethyleneimine as affinity surface	Plasma	0.2 U/mL	Lower detection limit than that found for previously cited techniques	[149]
	Opioid	Morphine	Immunoassays using polyclonal antibodies from New Zealand rabbits	Urine	762–24,4000 pg/mL	This technique enables the sensitive and specific quantification of different opioids such as heroin and morphine	[150]
**LSPR**	Anticancer	MTX	LSPR with functionalized gold nanoparticles with folic acid (FA-AuNPs)	Plasma	155 nM	This technique provides a new index for quantifying this drug by this type of biosensor	[127]
	Antibiotic	Tobramycin	Transmission localized surface plasmon resonance (T-LSPR), using DNA aptamers	Serum	0.34 µM	This modification enables the direct detection (without using labels) of a small molecule in a complex matrix	[155]
	Anticoagulant	Megalatran	LSPR integrated with a microfluidic chip	Solution buffer	0.9 nM	A pioneering study regarding the use of enantioselective biosensors	[141]
	Anti-arrhythmic	Digoxin	LSPR with gold nanoparticles	Solution buffer	2 ng/mL	This device enables the direct low-cost detection of digoxin, as well as being a device that is easy to make and use	[160]
**SERS**	Anticancer	5-fluorouracil	SERS with silver nanoparticles	Saliva	150 ng/mL	This study provides a great opportunity since it enables one to quantify a highly toxic drug with genetic variations in its metabolism	[162]
	Antibiotic	Ampicillin	SERS with silver on nanoparticles using hydroxylamine—HCl	Solution buffer	27 ng/mL	This technique has been compared to LC/MS, with greater sensitivity. It provides an index for quantifying drugs with this type of device	[163]
		Penicillin G			29 ng/mL		
		Carbenicillin			30 ng/mL		
		Penicilloic acid			28 ng/mL		
